# The work, goals, challenges, achievements, and recommendations of orphan medicinal product organizations in India: an interview-based study

**DOI:** 10.1186/s13023-019-1224-0

**Published:** 2019-11-04

**Authors:** Mohua Chakraborty Choudhury, Gayatri Saberwal

**Affiliations:** Institute of Bioinformatics and Applied Biotechnology, Biotech Park, Electronics City Phase 1, Bengaluru, Karnataka 560100 India

**Keywords:** Rare disease, Orphan drug, Orphan medicinal product, Orphan medicinal product organization, Policy, India

## Abstract

**Background:**

Orphan medicinal products (OMPs) are intended for the diagnosis, prevention, management or treatment of rare diseases (RDs). Each RD affects only a small fraction of the population, and therefore, historically, industry hesitated to undertake relevant research and development (R&D). In response, the governments of many countries came up with orphan drug policies and RD policies which were hugely successful in incentivizing companies to do so. In India, in the absence of any such policy until recently, there are very few organizations involved in RD R&D.

**Objectives:**

We wished to understand (i) the OMP Organizations’ (OMPOs’) areas of work and the nature of their work, (ii) their goals, (iii) the challenges they faced and how they were overcoming them, (iv) their achievements, and (v) their recommendations to the government to help their R&D, their success as commercial entities (where applicable), and patients’ access to their products or services.

**Results:**

Ten of the 14 OMPOs are companies, whereas four are not-for-profit organizations. Almost all of the OMPOs are heavily into R&D. Six have already made their products or services available to patients. Four plan to out-license their products after the pre-clinical phase or phase 1 trials, eight plan to cater to patients directly and two of the OMPOs have been established only recently and thus do not yet have any product or service to offer patients. Nine OMPOs import about 90% of the components in the production process, which comprises either capital or recurrent expenditure. For most, locally manufactured alternatives are not available or are of inadequate quality. Most of the OMPOs have had productive collaborations with local or foreign academics or hospitals for R&D, animal efficacy studies, clinical trials or providing services to patients. The main challenges for the OMPOs are the lack of adequate funding, supportive government policies, and a conducive ecosystem.

**Conclusions:**

These OMPOs are pioneers in their respective fields in India, and despite the challenges, have achieved new levels of innovation. With suitable government policies, they could scale up and provide relevant products and services to the large number of RD patients in the country whose medical needs are largely unmet.

## Background

Rare diseases (RDs) are debilitating medical conditions that affect a small percentage of the population. Research and development (R&D) to treat such diseases was long neglected by the pharma industry because of the likelihood of poor commercial outcomes. However, many countries have implemented policies that incentivize industry to develop ‘orphan drugs’ (ODs) to treat RDs [1]. The United States (US) was the pioneer, and enacted the Orphan Drug Act (ODA) in 1983 [2]. Several countries followed suit [3]. European Union legislation in 2000 covered a wider range of orphan medicinal products (OMPs), which included any product intended to diagnose, prevent, manage or treat RDs [4].

Orphan policies have been highly successful in driving RD R&D, and this has benefited both patients and the industry [5, 6]. Since 1983 the US Food and Drug Administration (USFDA) has awarded an orphan designation to 4905 products in-development, 771 of which have received marketing approval [7]. This has also impacted the overall landscape of innovative drug development [8, 9]. Over one-quarter of all new molecular entities (NMEs) that were approved from 1983 to 2017, inclusive, in the US had an orphan designation and in 2018, 34 of 59 approved drugs did [5, 10]. ODs also bring significant financial returns to the pharmaceutical industry. Worldwide OD sales are predicted to grow at a compound annual growth rate (CAGR) of 11.3% from 2018 to 2024, which is double the rate predicted for non-orphans [11]. Furthermore, ODs are expected to capture 20% of worldwide prescription sales by 2024 and to reach revenues of $262bn [11]. In most countries, OD policies are complemented by RD policies that have empowered patients with awareness, access to treatment and healthcare coverage [12]. A review of the policies of 35 countries showed that 33 countries provide reimbursement for ODs [13]. This has contributed to the sustainability of the industry by ensuring that patients can access these drugs despite their high prices.

In India, approximately 70 million patients suffer from an RD [14]. The prohibitive cost of any available OD, the absence of insurance coverage for most patients, delayed diagnosis and the lack of awareness of RDs and their management among doctors are some of the important challenges faced by these patients [15]. After much pressure from advocacy groups, in February 2017, the Government of India (GoI) announced The National Policy for Treatment of Rare Diseases (NPTRD). However, this was put in abeyance in November 2018 [16]. NPTRD primarily focused on making existing treatments available to patients through government funding. This approach has three major shortcomings: (a) providing treatment at the global price makes it impossible, financially, to cater to such a large patient pool; (b) many of the ODs, based on precision medicine, have been developed for a different population, and might not be effective for Indian patients [17]; and (c) some RDs may be more prevalent in India and need more urgent attention [18]. Further, in India, till recently, there was no official definition of RDs or ODs. However, the Central Drugs Standard Control Organization, which is the national regulatory body for Indian pharmaceuticals and medical devices, recently announced the New Drugs and Clinical Trials Rules, 2019 (NDCTR), which came into effect on 25 March 2019 [19]. These rules, for the first time, defined an OD as “a drug intended to treat a condition which affects not more than five lakh [5,00,000] persons in India”. It also provided three key incentives for the approval of ODs: (i) ODs which have been approved and marketed in certain other countries, may not require local clinical trial data to be submitted along with the application to import the drug into India. (ii) candidate ODs can avail of an accelerated approval process. (iii) the application fee for the conduct of clinical trials for ODs may be waived.

In the absence of policies for RDs, ODs or OMPs until recently, very limited RD R&D has taken place in India. Although, there have been studies of patient advocacy groups in India [14, 15], the Indian RD industry has not been studied so far. In many countries, such studies have provided valuable insights for policy-makers [3, 20, 21].

Therefore, for this study, we identified organizations that are involved in the development of products or services that aid in the diagnosis, prevention, management, or treatment of RDs. Collectively, we have classified these organizations as Orphan Medicinal Product Organizations (OMPOs). The study’s aims were to understand (i) the OMPOs’ areas of work and the nature of their work, (ii) their goals, (iii) the challenges they faced and how they were overcoming these, (iv) their achievements, and (v) their recommendations to the government to help their R&D, their success as commercial entities (where applicable), and patients’ access to their products or services.

These OMPOs are diverse both in terms of the RDs they cater to, and the products or services which they offer, or plan to offer. However, their experiences are a reflection of the Indian RD industry overall.

## Methodology

We have used a qualitative case study research methodology that has been used by others to study the healthcare industry [22, 23]. The reporting of the study was assessed using the Consolidated Criteria for Reporting Qualitative Research (COREQ) checklist, which helped to assesses the completeness of the reporting of the study [24].

### Researcher characteristics and reflexivity

GS: proposed the study. MCC: performed and analyzed the interviews. MCC and GS: wrote the manuscript and approved the final version. GS is an academic researcher and MCC is a post-doctoral fellow working with GS. Both have doctorates in the biological sciences. MCC and GS recently published a similar article based on interviews with RD patient advocacy groups [15], and GS has previously published other interview-based papers [23, 25]. Both GS and MCC are female.

### Ethics, consent, and permission

The study protocol was approved by the Institutional Ethics Committee of the Institute of Bioinformatics and Applied Biotechnology, Bangalore, India. Informed consent was obtained from each interviewee by requesting the person to respond positively to an email invitation. Each interviewee also signed an informed consent form.

An information sheet was provided to all the interviewees, which listed (i) the researchers’ affiliation, (ii) the source of funding, (iii) the study objectives, and (iv) the intention of publishing the findings in an academic journal. We subsequently obtained permission to use (i) the names of the interviewees and their organizations, and (ii) specific information from the interviews in the intended publication, with attribution. However, the interviewees’ approval of the entire manuscript was not sought.

### Data collection

The inclusion criteria for selecting an organization for the study were as follows: (i) The organization should have, or have in development, some product or service for RD patients. (ii) The organization should have an Indian origin, and preferably be registered in India. We sought out every OMPO that we could find, and requested almost all of them to be part of the study. The only exceptions were a few which were involved in genetic or genomic diagnostics, since that work was already well represented, and we wished to avoid an over-emphasis of one area. This was irrespective of the nature of the organization (company or non-profit), product or service being developed, or maturity of R&D (in development or marketed). As such, although the sample is small, it is merely a reflection of the nascent nature of RD R&D in India, especially in the corporate world.

We selected 20 such OMPOs through purposive sampling and invited the founding members or another key person of the organization to participate in the study. Some declined to participate, but did not mention their reason to do so. Others did not respond to the interview request within the study timeframe and were therefore excluded. 14 organizations agreed to participate: (i) Aten Porus Lifesciences (APL); (ii) Center for Drug Discovery (CDD) (iii) Dystrophy Annihilation Research Trust (DART); (iv) Eyestem; (v) Genes, Repair and Regeneration at Ophthalmic Workstation (GROW) at Narayana Nethralaya (NN) Foundation; (vi) Genetico; (vii) Genique Lifesciences (GLS); (viii) Genomics for Understanding Rare Diseases - India Alliance Network (GUaRDIAN), a collaborative program of the Institute of Genomics & Integrative Biology, an institute of the Council of Scientific and Industrial Research (CSIR); (ix) Indus Biotech (IB); (x) MedGenome; (xi) Pristine; (xii) Redcliffe Life Sciences (RLS); (xiii) Specialized Mobility Operations and Innovations (SMOI); and (xiv) TerraBlue XT (TBXT). CDD (set up by World Without GNE Myopathy (WWGM)) and Genetico were established in 2019, and are too young to have significant achievements. However, these organizations were included as they are focussed on RD R&D. Genetico is developing a unique range of services, and CDD is catalyzing RD R&D in other organizations. It will therefore be interesting to track their progress in future. Although, as mentioned later, these two organizations did not contribute very significantly to the interviews, they have a different strategy (CDD) or type of service (Genetico), and therefore add to the richness of the OMPO landscape in India.

Although the OMPOs were very diverse, we achieved data saturation in the results, in the following sections: products in development, production process details, collaborations, technical know-how, government support, intellectual property issues, and challenges.

Only three interviewees were known to the study team prior to the start of the study, and other OMPOs were identified using a snowballing method. The interviewees were contacted by e-mail, and a preparatory call was set up to brief them on the objectives of the study, purpose of the interview, researchers’ interest and to confirm their participation. For those located in Bangalore, and for some of those based in Delhi, the interviews were conducted face-to-face, at a location convenient to the interviewee. The other interviews were conducted by phone or Skype. The interviews lasted between 24 min and 116 min, and on average took 52 min. MCC conducted all the interviews between June 2018 and April 2019, inclusive. In some cases, the interviewees were subsequently contacted for clarification or further information. Usually, one person was interviewed from each organization and no one else was present besides the interviewee and the interviewer. However, in the case of GUaRDIAN, the primary interviewee connected the interviewer with a colleague for details of the ‘Products in development’ section of the interview schedule. Most interviewees responded as representatives of their organizations, not in their personal capacities (Table 1).

**Table 1:** Details of the OMPOs and the interviewees

Organization; URL; Type of organization; Year of establishment^a^	Interviewee; Role of the interviewee in the organization	Category of work	Vision, mission and goals^b^
Aten Porus Lifesciences (APL) (http://www.atenporus.com/) Company; 2014	Dr. Aditya Kulkarni^c^ Founder and Chief Scientific Officer	Treatment: Drugs	Drug discovery for diseases with unmet medical needs in India.
Dystrophy Annihilation Research Trust (DART) (https://dartindia.in/) Not–for–profit Trust; 2012	Dr. Arun Shastry^d^ Chief Scientific Officer	Treatment: Genomic interventions	Research for affordable therapies for muscular dystrophies. Helping families with diagnosis, counselling and management of the disease.
Eyestem (https://www.eyestem.com/) Company; 2015	Dr. Jogin Desai^c^ Founder and Chief Executive Officer (CEO)	Treatment: Cell therapies	Cell therapy that is scalable and affordable.
Genique Lifesciences (GLS) (https://www.genique.co/) Company; 2018	Mr. Abhishek Das^c^ Founder and CEO	Diagnostics: Genomic-based diagnostics	To make genetic reporting easy-to-understand for clinicians and patients.
Genes, Repair and Regeneration at Ophthalmic Workstation (GROW) (https://www.narayananethralaya.org/grow-research-lab/) Research laboratory based in a not-for-profit hospital; 2013	Dr. Arkasubhra Ghosh^d^ Director, GROW	R&D	Discovery research using patient tissue, targeted towards the following: understand the genetic basis of diseases; modelling of diseases to understand the function of genes in pathological processes; and development of gene therapies and clinical grade vector production for trials in humans.
Genomics for Understanding Rare Diseases - India Alliance Network (GUaRDIAN) (http://guardian.meragenome.com/) Not–for–profit collaborative research program hosted by a government research institute; 2013	Dr. Sridhar Sivasubbu^d^ Principal Scientist at CSIR IGIB & Co-founder of GUaRDIAN	R&D	The vision of GUaRDIAN is to establish a unique collaboration framework in healthcare planning, implementation, and translation in the specific area of rare genetic diseases. The program proposes to apply genomics for the systematic characterization and diagnosis of rare genetic diseases in India with the aim of developing affordable and accessible genomic-based diagnostics available to people with rare diseases.
Indus Biotech (IB) (http://www.indusbiotech.com/) Company; 1998	Mr. Sunil Bhaskaran^d^ Founder and Managing Director	Treatment: Drugs	To develop botanical drugs for RDs and seek approval from the USFDA, under the orphan drug category.
MedGenome (https://www.medgenome.com/) Company; 2013	Dr. V L Ramprasad^d^ Chief Operating Officer and Lab Director	Diagnostics: Genomic-based diagnostics	Its vision is to enable precision medicine. Its mission is to provide comprehensive molecular testing solutions that helps make an informed decision for drug discovery and disease management. It is the market leader in genetic diagnostics in India and one of the highest throughput NGS sequencing labs in South Asia
Pristine (https://pristineorganics.com/) Company; 2004	Ms. Shruti Kumbla^d^ Senior Nutritionist	Management products: Nutritional products, IEM foods	To provide medically-appropriate nutritional food
Redcliffe Life Sciences (RLS) (https://www.redcliffels.com/) Company; 2017	Mr. Ashish Dubey^d^ Founder and CEO	Diagnostics: Genomic-based diagnostics	DNA based diagnostics for mother and child diseases
Specialized Mobility Operations and Innovations (SMOI) (http://smoi.org/) Company; 2012	Dr. Padmaja Kankipati^d^ Director	Management products: mobility aids	Providing services related to customized wheeled mobility solutions based on the user’s physical, functional and environmental needs, and making world class technology accessible to patients through innovation.
TerraBlue XT (TBXT) (https://www.teblux.com/) Company; 2016	Ms. Rajlakshmi^c^ Borthakur Founder and CEO	Management products: wearable medical devices	To create Internet of Things (IoT)- and Artificial Intelligence (AI)- based solutions and wearable medical devices for neurological disorders.
New Organizations			
Center for Drug Discovery (CDD) (http://gne-myopathy.org) Not–for–profit Trust; (CDD was launched in 2019)	Dr. Sudha Bhattacharya^d^ Founder	R&D	Mission is to collaborate with various research institutes across the country to fund research leading to the development of therapies for rare diseases, specifically GNE Myopathy
Genetico (Website under construction) Company; 2019	Mr. Arjun Gupta^d^ Founder	Management products: Genetics and genomics based software technologies.	Genetico is a genetics and genomics company focused on discovery science and reducing time to diagnosis in the area of rare diseases. Our goal is to enable cost effective diagnostics and therapeutics in emerging countries.

^a^ Year of establishment refers to the year of registration of the organization, or launch of the program in the case of CDD, GROW, and GUaRDIAN

^b^mission, vision and goals are not well defined for all of the OMPOs, and we have include any relevant information provided by the interviewees

^c^The interviewee responded in personal capacity

^d^The interviewee responded as a representative of the organization

The interviewer got back to the interviewees for approval of (i) the interview transcript, (ii) extracts of the manuscript that needed their consent for inclusion in the manuscript, and (iii) using the interviewees’ and their organizations’ names for information that is included in Additional file 2 and as specific quotes in the paper, with attribution. The interviewees’ approval of the entire manuscript was not sought. We took a few months to complete the interviews and analyze the data, and by that time the government had announced some policy changes. When we sent the interviewees their portion of the data that was intended to be included in the manuscript, their clarifications sometimes addressed these regulatory changes. We have used organizations’ names instead of interviewees’ names in the paper, although the interviewees are identified in Table 1. Further, the research programs (GROW and GUaRDIAN) of two institutions are referred to as organizations for convenience. The comments quoted in the paper were edited for clarity, before obtaining the concerned interviewee’s approval for use.

We conducted semi-structured interviews using a pre-designed interview schedule (Additional file 1) which inquired about the work, goals, challenges, achievements, and recommendations of the OMPOs. The interview schedule was pilot tested with five non-interviewee researchers, and this led to some revisions prior to the initiation of the study.

### Data analysis

Field notes were made during the interview and the interviews were also audio-recorded. The recordings of the interviews were transcribed verbatim for analysis. No software was used for analysis. Instead, the interviews were parsed using a python script to code and extract information. The transcripts were analyzed using a thematic analysis which was reviewed by both the members of the research team. The interview was based on a largely closed-ended interview schedule. The themes were derived based on specific research questions and thus largely overlapped the topic-guide of the interview schedule. The interview schedule covered eight major areas, as follows: (i) the organization; (ii) products or services; (iii) the production process; (iv) collaborations; (v) technical know-how; (vi) government support; (vii) intellectual property; and (viii) challenges.

We used a qualitative content analysis method to analyze the data and no statistical techniques were used.

## Results

Basic information about the 14 OMPOs, and the interviewees representing them, is provided in Table 1. The two organizations established in 2019, CDD and Genetico, are primarily covered in the section ‘Organizational details’. Elsewhere, the analysis relates to the 12 other OMPOs, unless otherwise specified.

### Organizational details

All the 14 OMPOs are engaged in the R&D of products or services for RD patients, although in the case of CDD the engagement is indirect. Four of them provide drugs or therapies, three diagnostics, four management products and three are engaged in R&D (Table 1). Ten are companies, whereas four are not-for-profit organizations.

IB and Pristine were founded in 1998 and 2004, respectively, whereas all the others were set up from 2012 onwards. Most of the companies are in the start-up phase. In terms of funding, eight OMPOs have been self-funded. In addition, CDD and GROW were initially funded by their parent organizations (WWGM and NN respectively); Eyestem and IB by angel investors; and MedGenome and RLS by venture capitalists. GUaRDIAN is entirely supported by the government. Some OMPOs have also subsequently received financial support from a GoI program that supports entrepreneurship.

Eleven OMPOs started with a focus on a particular RD while the remaining three were initially engaged in other areas of work (Additional file 2). For all of them, the primary motivation for starting work in the area of RDs was the lack of treatment or management options and also the lack of research on RDs in the country. Furthermore, the founders of CDD, DART and TBXT were parents who wished to develop a treatment for their child’s disease.

Although each organization had a unique mission, the primary goal of each of them was to address the unmet medical needs of the relevant patients. Most of the OMPOs reported a lack of prevalence data for their concerned diseases which made it challenging to carry out a market survey to gain insight into the demand for their product in the country. Nevertheless, most of the OMPOs, especially the companies, did carry out formal market research, and a couple of them did informal surveys. (Additional file 2). With time, some of the OMPOs have pivoted from their original product line. IB started a nutraceutical segment in addition to its drug development segment, which was expected to help them generate revenue earlier. Similarly, SMOI recently restructured, and increased its focus on education, R&D and advocacy. It has stopped importing mobility devices and has instead collaborated with a product partner to provide the relevant products.

### Product or service

The products and services of each OMPO are outlined in Table 2. As mentioned earlier, we primarily focus on the 12 older OMPOs. They are based on different technologies as follows: (i) APL and IB: small molecule drug discovery; (ii) DART, GROW, GUaRDIAN: genomic and molecular technologies; (iii) Eyestem: cell therapy; (iv) GLS, MedGenome and RLS: genomic diagnostics; (v) Pristine: food processing technologies; (vi) SMOI: primarily services related to mobility devices and (vii) TBXT: IoT and AI technologies. Further details are available in Additional file 2.

**Table 2:** Details of the products, services or R&D activities of the OMPOs

Organization	Products/Services
APL	APL has discovered drugs for Niemann-Pick disease (NPD) and focal segmental glomerulosclerosis (FSGS). It is also working on drugs for non-RD diseases. Two drugs are in the early discovery stage, one for non-alcoholic steatohepatitis (NASH) and the other for obesity. Further, it is looking at other drugs for metabolic syndrome.
DART	Exon skipping therapy for muscular dystrophy.
Eyestem	Cell therapy for ophthalmic diseases such as retinitis pigmentosa (RP) and macular degeneration (MD)
GLS	Carrier screening test that includes 10,000 pathogenic genomic variants which cover 1700 diseases.
GROW	Discovery-based clinical applications and gene therapy for RP.
GUaRDIAN	R&D for molecular genetic diagnostics; identifying novel markers specific to a disease and population; education in clinical genomics.
IB	Botanical drugs: 3 are in the pipeline: 1) a drug for Levodopa-induced dyskinesia (LID) 2) drugs for Huntington’s disease 3) drugs for Sumatriptan-resistant migraine patients.
MedGenome	There are three business segments: (i) Research services: mainly for pharmaceutical companies and research organizations which outsource work. (ii) Genetic diagnostics: Primarily focused on monogenic diseases and oncology, as India has a huge population suffering from these diseases. It focuses on actionable mutations such as from lung, colorectal, and breast cancer. Identifying predisposition mutations and individuals-at-risk in families. Helping them to manage the disease, and increasing surveillance. (iii) Data analytics and developing new databases. It has a fairly large bioinformatics team which develops algorithms and pipelines, and helps create unique databases. It works with clients across the world on customized solutions, or licensing out technologies that it develops. It has a lab in the US which caters to the pharma and biopharma industry. The first and third services are mainly handled by the US lab, whereas the second service is mainly handled by the India center.
Pristine	Broadly, products are divided into 5 segments, of which segment (v) is for RD patients: i) fortification of rice, flour, oil, milk; ii) pre-mixes of nutritional supplements; iii) animal feed pre-mixes for the poultry, equine and aquaculture industries; iv) Organic staples: Rice, millets, flours, oil seeds, cold pressed oil and organic infant food; (v) Food for special dietary uses; and (vi) Nutritional supplements for specific disease conditions. These supplements are categorized by the Food Safety and Standards Authority of India (FSSAI) as ‘Food for special medical purpose’. They are sold under the brand name ‘Balance Metanutrition’, and are for IEMs, diabetes, autism, cancer, Alzheimer’s.
RLS	DNA-based diagnostics for maternal and child health conditions such as NIPT, microarrays, single gene disorders, clinical exome, whole exome, whole genome.
SMOI	(i) Services related to mobility aids and accessories (ii) A low-cost mobility device is in development
TBXT	IoT- and AI-based wearable medical devices for neurological disorders. The device for epilepsy can 1) predict the occurrence of an epileptic attack; 2) assess the efficacy of medicine; and 3) predict any serious, potentially fatal, attack.
New Organizations	
CDD	Collaborate with various research institutes across the country to fund research leading to the development of therapies for rare diseases, specifically GNE Myopathy.
Genetico	Software and database management system to assist geneticists in diagnosing RD patients. Some of the strategies include matching patients with similar clinical profiles to help achieve diagnosis and knowledge about specific RDs.

As such, the OMPOs cover a wide range of products that cater to different groups of RD patients. All 12 OMPOs have products in development, with eight having products in the pre-clinical phase. So far, only six OMPOs’ products or services have reached patients, and most of the products aimed at therapeutic treatment of RDs have not yet done so. APL has out-licensed two drugs for RDs related to lipid metabolism whereas IB has brought nutraceutical products to the market. Drugs for around five RDs are in various stages of development. DART’s exon-skipping therapy for Duchene muscular dystrophy is in clinical development. Eyestem and GROW are developing therapies for retinitis pigmentosa (RP), the former a cell therapy and the latter a gene therapy. However, most of the management products have already reached patients, or are in late stages of development. Pristine produces special dietary supplements for 17 Food Safety & Standards Authority of India (FSSAI)-identified disorders. SMOI provides services related to the assessment of wheelchairs and other mobility aids, for a variety of RD patients with mobility disorders. TBXT is running a clinical trial for its wearable medical device, which can monitor epilepsy and other neurological disorders.

Four OMPOs plan to out-license their products after the pre-clinical phase or phase 1, whereas the remaining eight plan to reach out to patients directly.

With the exception of the diagnostic companies, most of the OMPOs do not have any competing products in the Indian market. The products and services offered by these organizations are usually more affordable than the global alternatives. Various strategies such as local sourcing of components, innovative design, lower cost of manufacturing, and a reduced profit margin help to reduce prices (Additional file 2).

### Regulatory approvals

The regulatory approvals received by each OMPO have been detailed in Additional file 2. Only five have received regulatory approvals for their products so far, and four of these are from India. In seven cases, no regulatory approval is required. Most OMPOs wish to take their products to other countries, and four of them are applying for approval from both Indian and foreign drug regulatory agencies. The costs of filing an Investigational New Drug Application with the USFDA are very high. However companies such as IB are inspired by the OD policy of the US, which significantly reduces the cost of drug development for a small start-up company. IB is seeking USFDA approval for its products under the botanical drugs category. Its products are prepared from food-chain raw materials that are categorized as ‘Generally recognized as safe’ (GRAS) in the US.

Management products require simpler and less expensive regulatory approval. Pristine is a food-based company which only needs approval from FSSAI. Diagnostic companies such as GLS, MedGenome and RLS need National Accreditation Board for Testing and Calibration Laboratories accreditation for quality and technical competence assurance of their services. Both MedGenome and RLS are Pre-Conception and Pre-Natal Diagnostic Techniques-certified laboratories. Additionally, MedGenome has accreditation from the College of American Pathologists, which has helped in its global outreach.

### Production process details

Nine OMPOs import about 90% of the components in the production process, which comprise 90% or more of the cost of production. These costs may be capital expenditure, recurrent expenditure, or both. For most, a locally manufactured alternative has been unavailable or of inadequate quality. All of the interviewees agreed that substituting with high-quality products, if locally available, would reduce the price significantly, since these imports attract custom duties of about 25%. Furthermore, most of the OMPOs had huge setup costs as they had to import most instruments, and several of them had spent over 100 million rupees to date. These components and instruments are also used by many other organizations in the country, and thus, local manufacturing could cater to the large domestic demand, and significantly reduce production costs.

### Collaborations

Ten of the OMPOs have had productive collaborations with local hospitals and clinicians and five of them have also collaborated with local academics (Additional file 2). Some of these collaborations have been for clinical trials.

Nine OMPOs have an active collaboration with a foreign academic for basic scientific research and advice, as detailed in Additional file 2. Additionally, four of these nine OMPOs, that is APL, DART, Eyestem and GROW, have partnerships with international academics for animal efficacy studies. Most of the foreign collaborations were established through personal academic contacts. Eight of the nine mentioned that they did not face any major challenge in establishing these collaborations. International collaborations have facilitated the exchange of knowledge with the global RD R&D community and have helped to shape the RD R&D sector of India. A couple of organizations such as DART and GROW have immensely benefitted from such collaborations by the training of personnel at leading global research centers, and by scientific advice.

### Technical expertise

The OMPOs have not spent significant funds to gain technical knowledge. Most of the founders, or key people in R&D, have returned from Western nations with post-graduate degrees or relevant industry experience. This has helped to accelerate the start of RD R&D in India, at global standards, and reduced the time required to train local scientists. The technical know-how of all of the OMPOs was developed in-house, sometimes with formal or informal scientific advice from other organizations. Most of the companies have had a consistent technical strategy, with suitable modifications, over the years. Eight of them did not face any challenge in developing technical know-how. However, two of the OMPOs mentioned that they did face some challenge in this endeavour due to the lack of expertise in certain domains (Additional file 2).

### Government policies and support

Some of the OMPOs have benefited from supportive government policies, as detailed in Additional file 2. Three OMPOs have received support from the government through a Biotechnology Ignition Grant, which is a biotech funding program for young startups and (potential) entrepreneurs across areas, for their proof-of-concept studies. Others have received project-based grants from other government programs. In addition, they have been able to obtain various tax exemptions after being recognized as R&D organizations by the Department of Scientific and Industrial Research, GoI. GUaRDIAN is entirely supported by the government.

Driven by the activism of patient groups such as MERD-India, and supported by companies such as Pristine, FSSAI launched a program, Diet4Life, to address the lack of specialized diets for patients with inborn errors of metabolism (IEM). This has been done in partnership with professional organizations, healthcare professionals and companies. FSSAI has chosen five companies to address the issue, which are Abbott Nutrition, Danone (Nutricia), Mead Johnson Nutrition, Nestle and Pristine, of which Pristine is the only Indian company.

Another instance of policy change driven by activism was seen in the reduction of the Goods and Service Tax (GST) rate for wheelchair accessories. In 2017, GoI replaced many indirect taxes with the system of GST slabs. A GST of 5% was imposed on wheelchairs but 28% on wheelchair parts and accessories. Although equally clinically relevant, these parts and accessories were considered luxury items. Following this, SMOI, along with several disability rights groups, advocated for a revision of GST rates. In 2019, this resulted in a reduction of the GST levied on wheelchair parts and accessories to 5%. Thus, whereas there have been government programs that benefit the OMPOs, patient activism has played an important role in effecting policy changes.

CDD suggested that (i) the contribution of Corporate Social Responsibility funds towards research be encouraged, (ii) RD research funding raised by patient organizations be matched with grants from the Government, and (iii) RD patient groups be included in relevant policy-making bodies of the Government. GoI requires that a charitable group be registered under the Foreign Contribution Regulation Act (FCRA) to legally receive contributions from any donor outside India. CDD mentioned that it is unable to receive funds from international organizations as it does not have FCRA approval, and therefore suggested that RD organizations should be exempt from such requirements.

### Intellectual property (IP) issues

There are no separate benefits for Indian companies pursuing RD R&D. However the OMPOs have benefitted by protecting their IP. Eight OMPOs have protected their products with patents and one with a trademark for its brand name (Additional file 2). Eight have filed patent applications in India and two have also filed in other countries. Most of the OMPOs have neither in–licensed nor out–licensed IP. However, APL has out-licensed two drugs, and MedGenome has in-licensed a diagnostic test and out-licensed several pieces of software. GUaRDIAN mentioned that they out-license their products and technologies without any charge, so as to keep the ultimate price for patients as low as possible. Non-exclusive sharing of IP rights also ensures lower price points. None of the OMPOs have faced any challenge related to gaining, protecting or defending their IP. Also, none of them have encountered IP theft in India.

### Challenges

The organizations of this study have different domains of expertise and a wide range of activities. As such, they have different challenges, which are detailed in Additional file 2. However, they do face common challenges which are summarized below:

(i) Inadequate funding: The OMPOs faced a major struggle in raising funds for setting up, scaling-up manufacture or marketing. APL highlighted the need for more opportunities for start-up and scale-up funds, and grants for basic research in academia. TBXT said “We were offered 2 million dollars by the Australian government but I declined, as it would require a transfer of IP from India. However, now I am facing difficulties in taking off because of the lack of government support here.” GLS mentioned that “Barring a few, existing genetic diagnostic companies have not developed successful business models. This has discouraged investors from funding similar organizations.”

(ii) Lack of supportive government policies: All OMPOs have faced challenges on this score. Interviewees mentioned that because of lack of initiatives such as an OD or RD policy, such drugs do not get any special designation or priority for approval by the DCGI. Therefore, investors recommend applying for USFDA approval. DART also emphasized the need to formulate policies related to ‘right–to–try’ and ‘compassionate use’, whereby a seriously ill patient, with no treatment options, could access an unapproved drug. MedGenome pointed out that because of lack of government support, such companies often lose out in international competitions. It suggested that the government should take steps similar to those by the Chinese government to incentivize such companies. For example, OMPs in India do not receive tax breaks or exemptions from customs duties. Lack of proper regulations and transparent guidelines add to the problem. Furthermore, officials may be poorly trained and this leads to the poor implementation of existing policies. GROW suggested that the government should fund and encourage collaborative national projects such as a national program for gene therapies, so that investigators in different organizations could work on parts of a larger program.

(iii) Need for a supportive ecosystem: Most of the OMPOs working on genomics data reported that since genomics is not included in the medical curriculum in India, the training of clinicians, and convincing them to consider genomics-based diagnostics, is an important unfinished task. Furthermore, most genomic assays are either unaffordable or inaccessible to most Indian patients. Supporting industries, such as those of biochemicals and other consumables need to grow to ensure the sustainable growth of this sector. Likewise, insurance companies need to cover OMPs. TBXT said that “Because of the lack of local manufacturing units, we had to partner with manufacturers from Taiwan and China. This led to inconvenience and added to the cost because of visa charges, travel costs, regulatory issues and time”. SMOI emphasized the need to establish quality control standards for products and services. Furthermore, there is a lack of awareness among doctors and end-users about the need to use customized mobility devices. MedGenome pointed out that the price-sensitive market is another big challenge; each product and service has to be developed keeping the ultimate price in mind.

(iv) Lack of facilities for animal studies: A few OMPOs mentioned the shortage of well-equipped animal facilities required to support discovery research in the country. DART said that “The required mouse-model is unavailable and importing a single animal costs around USD 320. Therefore the Contract Research Organizations (CROs) in India only do toxicity studies. We had to collaborate with Leiden University to carry out animal efficacy studies.” GROW recommends a state–of–the–art animal facility in the country, with facilities that could be rented by various research programs.

(v) Absence of data: All the OMPOs reported a lack of patient data from India, which posed a huge challenge for their R&D and their regulatory filings. Pristine mentioned a lack of market information and prevalence data, and poor networking among health professionals who care for patients with IEMs. This made it difficult for the field force to identify relevant doctors, especially in remote areas, to make them aware of Pristine’s products. GROW mentioned that there is a dearth of data related to disease natural history, morphometry, family history and genetic information. TBXT also faced a challenge due to the lack of physiological datasets for epilepsy patients in India.

(vi) Challenges in establishing collaborations: Nine of the OMPOs had productive collaborations with local academics and hospitals. However, APL had difficulties in finding a local collaborator because of a dearth of subject experts and translational expertise in the country. Furthermore, it mentioned that investors lack confidence in Indian academic partners. Most of the OMPOs reported that they did not face any challenges in establishing international collaborations. However, TBXT faced challenges because of the lack of international credibility of Indian entrepreneurs, and it required extra effort to convince potential collaborators.

### Recommendations

As a part of the interview schedule, the OMPOs were asked about the kinds of changes they would like to see in government policy that would help their R&D, or business, and also those that would help their patients (Additional file 2). Some of the key recommendations made by most of the OMPOs are listed below:

(i) The government should enact a national policy to facilitate the local development of OMPs. It should include incentives similar to those of other countries, such as tax incentives, enhanced patent protection and marketing rights, clinical research subsidies, and creating a government-run enterprise to engage in relevant R&D.

(ii) NPTRD needs to be reinstated and implemented, to (a) enable access to diagnosis and treatment, (b) stimulate research, and (c) promote patient engagement in R&D and policy-making.

(iii) The government should provide healthcare coverage for all RD patients.

(iv) The government should enact and implement policies to support and protect related industries such as those of healthcare, biotech, and CROs, including those offering animal testing services, consumables and so on.

(v) Academia should engage in more translational research, and be more willing to collaborate with companies.

(vi) All the OMPOs supported the enactment of an OD policy.

Additionally, SMOI suggested that standards for services and products should be established. TBXT emphasized … “the training of personnel working in government agencies that are involved in implementing policy. These officers need to be sensitized to the start-up ecosystem.” MedGenome recommended that … “The Indian government should emulate the Chinese government on incentivizing, subsidizing, and liberally funding various sectors to create a supportive ecosystem.”

## Discussion

To summarize our findings, most of the OMPOs were formed from 2012 onwards. As such, their R&D is still largely in the early stages, although six have brought products or services to market. Even those at a later stage are struggling to stabilize or scale-up. They cover a wide range of products across the three major segments of RD care, that is diagnostics, treatment, and management, for different groups of RD patients. The OMPOs also belong to different industries such as biotech, medical devices, food and pharma. Although self-funding has been an important driver, institutional funding by angels, venture capitalists (VCs) and the government has played an important role. Several of the OMPOs have spent over 100 million rupees to date. Most of the organizations started work with a focus on RDs, although a few came to this area later. With the exception of the diagnostic companies, most of the OMPOs do not have any competing products in the Indian market. Most of the products and services offered by these organizations are more affordable than the global alternatives. The cost could drop significantly if the high-quality products required for production, currently imported, were locally available. Only five OMPOs have received regulatory approvals, of which four are from India, and in seven cases, no regulatory approval is required. Most of the founders have developed their organizations’ technologies on their own, sometimes with formal or informal scientific advice from other organizations, thereby accelerating their R&D. Of those who received support from the government, most benefited by funding, and the rest through tax exemptions. Most sought greater support from the government through an OD policy, and through health coverage for patients. Eight OMPOs have protected their products with patents, with most of the filings being in India. Despite the heterogeneity of the OMPOs, they had many common challenges including inadequate funding, the lack of tailored government policies, the need for a supportive ecosystem, the lack of facilities for animal studies, and the absence of various kinds of data related to RDs. The OMPOs believe that a common OMP policy would address many of these issues. As listed above, the provisions made by the NDCTR relating to accelerated approval and waiver of application fees for clinical trials will benefit the five OMPOs which are in trials, or which hope to conduct trials.

We need to place the rise of these OMPOs in context. The pharma industry in India has evolved over the last 50 years or so. From 1972, for a few decades, product patents of pharmaceuticals were not recognized. This led to the rapid growth of the generics’ sector and today India is a global leader in this area [26]. In the 1990s, it made forays into discovery research, and since the early 2000s, there has been a rise in the number of biomedical and biotechnology firms [26, 27]. Although there was a surge in the development of ODs in the US after the passage of the ODA in 1983, India did not witness much interest in this field until fairly recently. In 2012, NATCO became the first Indian company to receive an FDA OD designation for one of its products [27]. So far, only two other Indian companies, Lupin and Regrow Biosciences, have received an OD designation in the US or in Europe for their products. One of them has also received marketing approval, from the European Commission [28, 29]. To the best of our knowledge, no other product from India has received an OD designation or reached the market in the West.

The slow growth of this sector is similar to that seen for drug-discovery in India in general. In the early 1990s it was thought that India would become the drug discovery powerhouse of the world [30]. However this did not happen, and of about 200 drugs that were in various stages of preclinical and clinical development in the country, only one innovative drug, Lipaglyn, reached the market [27, 31]. Subsequently, many Indian companies severely cut back, or abandoned, their R&D activities [27, 32]. Potentially, orphan product R&D could be a niche area for the industry to invest in, should the government provide suitable incentives. Globally, there is a call for the repurposing of drugs for RDs [33, 34], and these 200 compounds, of which 82 progressed to Phase 1 and 34 to Phase 2, could be revisited to test their efficacy for an RD [27]. Furthermore, over these 20 years, India has developed drug discovery expertise, that could be utilized by the OMP industry.

Globally, academia, smaller companies, and biotech firms have led the discovery and development of ODs [35, 36]. A similar trend is seen in our cohort, where most of the organizations are start-ups and 10 of them are biotechs. We also need to view the rise of these start-ups in the context of the general rise in entrepreneurship in India [37]. In recent years the GoI has initiated many programs to nurture innovation across sectors [38]. In the bio-medical sector, the efforts of the Biotechnology Industry Research Assistance Council (BIRAC), established by GoI, are the most notable. It has supported over 1000 entrepreneurs in the last 7 years, some of whom have brought products to market [39]. Thus, the emergence of the OMPOs coincides with a significant rise in biotech entrepreneurship in the country. All the OMPOs studied here have local roots, and started from scratch. Even as recently as the early 2000s, most innovative startups in India were spin-offs from multinational companies [23], and thus the OMPOs discussed here are part of a new breed of indigenous healthcare start-ups in India.

In India there is a general lack of awareness about RDs among various stakeholders [15]. This is evident from the fact that only 450 of the 7000 RDs recognized globally have been identified so far in India [40]. However, the last decade has seen an increase in activism, and of awareness, related to RDs in India, with the emergence of many disease-specific groups and umbrella organizations [15]. It is no coincidence that the development of OMPs picked up in India at about the same time. Notably, it was a high profile story when, following the market shortage in 2016 of D-Pencillamine, used for Wilson’s Disease, patient support groups successfully lobbied a local pharma company to manufacture a better alternative, Tirentine [41]. OMPOs in the study such as CDD, DART, and TBXT are directly related to patient activism since they were started by parents of affected children. Others such as Eyestem, GROW, GUaRDIAN, MedGenome, Pristine, and SMOI also support, or are involved in, patient advocacy. Formalization of patient participation in decision making would help to receive their insights in the early stages of policy formulation.

It is remarkable that despite the absence of an OD policy in India until recently, several organizations have been pursuing such research. Nine OMPOs are focused on making their products and services tailored to, and accessible to, Indian patients. Most of them would like to carry out their entire R&D in India, to ensure that their products are affordable and immediately available to Indian patients. However, due to inadequate funding to carry out clinical trials, four of the OMPOs plan to out-license their products after pre-clinical or phase 1 studies. This is unfortunate, since globally, when such products are acquired by multinational companies, their subsequent pricing makes them inaccessible to most Indian patients [42]. The lack of suitable incentives for the development of OMPs in the country has also led six of the OMPOs to eye international markets, and they are planning to apply to foreign regulatory agencies such as the EMA and the USFDA. Should they succeed in gaining such approvals, they will have access to lucrative markets, which will help fund further R&D. However, gaining such approval is an expensive process [43, 44] and such products may also become inaccessible to Indian patients. The government could help these companies gain international markets by funding such applications on the condition that they subsequently repay the support, or provide their products at affordable prices to Indian patients.

Furthermore, in the absence of prevalence data and government policies that aid access to OMPs, it is difficult to imagine a large market in India. Many Western nations have much better prevalence data and have enabled much higher pricing of OMPs, or policies that enable access, making them attractive markets for any OMP developed in India. However, as mentioned above, such a product may become unaffordable in India. Also many of the OMPOs have benefitted by protecting their IP. One of the authors (GS) published the findings of an interview-based study of 50 Indian biomedical start-ups in 2016. When asked, from a list of possibilities, the primary reasons for patenting, the most common responses were: (i) improve the valuation or image of the company, and attract VC or other funds; (ii) protect technology from imitation; and (iii) improve inter-firm deals. Possibly, similar reasons hold true for the OMPOs of this study.

One of the unintended consequences of the ODA has been the exorbitant pricing of these drugs in the US, and in other countries that implemented a similar Act [45]. While enacting an OMP policy, Indian policymakers should work to ensure patient access to the OMPs. Some of the ways this can be achieved are:

(i) India already has price control mechanisms for generic drugs and certain medical devices, and these could be tweaked to control the prices of novel OMPs [46]. However, in a recent notification, effective from 3 January 2019, GoI has exempted ODs from any price control [47]. This was done to incentivize local pharma to undertake innovation, and to make the Indian market more attractive for companies wishing to import ODs approved elsewhere. This move may enhance the availability of ODs, but it may negatively impact their accessibility.

(ii) In the past, the government has also tackled high drug prices by (a) restricting patent evergreening, by which an organization tries to extend the monopoly rights over a process or product whose patent is on the verge of expiry, by obtaining fresh patents based on minor changes to the protected process or product, and (b) through compulsory licensing, a provision by which the government permits an organization to use a patented process, or produce a patented product, without the patent-holder’s permission, while paying the latter an adequate amount [48, 49]. Such measures could be applied to OMPs as well.

(iii) Globally, the development of ODs by socially motivated, not–for–profit organizations has helped to facilitate access to more affordable ODs, as exemplified by the gene therapies of Genethon in France [50]. Four OMPOs in our study are similar organizations. The government should incentivize more such organizations to take up the development of OMPs, which would help to keep costs down.

(iv) The involvement of academia in early stage drug-discovery could significantly reduce the cost and risk for industry [51]. About 12 years ago it was noted that there was not much academia-industry collaboration in biotech in India [37]. However, in recent years there has been a rise in such collaborations, with BIRAC alone funding 115 projects run as industry-academia collaborations, and involving 88 academic institutes [52]. Also, the majority of incubators for biotech start-ups are either part of academic institutions or are hosted by them, and this helps to spur such collaborations [53]. GROW and GUaRDIAN are academic groups which have partnered with industry, and eight of the OMPOs which are companies have had partnerships with Indian academia. As seen in the biotech sector in general, the OMPOs’ collaborations with foreign academia and industry largely overcame the shortage of local subject matter experts or those with translational expertise [23, 54]. To enhance skill development in the country, the emphasis should be on enabling the creation and sustainability of support systems, as outlined above in recommendations by the OMPOs in the Results section.

(v) If patients are provided healthcare coverage, and they and the medical fraternity are empowered with more information, it will lead to an increase in the demand for such products, which would boost the OMPO sector. Moreover, since many RDs are life-debilitating, patients need life-long management with assistive drugs, foods, and devices [55, 56]. Many of these products are not manufactured locally, making them very expensive. Health coverage schemes should include such products as well.

The biggest achievement of the RD movement in India has been the inclusion of incentives for the OD sector in NDCTR. However, a comprehensive policy that covers all aspects of RD diagnosis, management, prevention and treatment is much needed. Such a policy should support and incentivize the OMP industry. Before NDCTR, the policies concerned with (i) disabilities, (ii) plasma, (iii) stem cells, (iv) medical devices and so on had a positive impact on RD patients and the OMP industry. However, all the OMPOs have faced challenges due to the poor implementation of these policies. Thus, emphasis should be laid on the effective implementation of policies related to OMPs. In earlier studies, four critical factors have been identified for the poor implementation of policies in any country [57]. The OMPOs in this study agreed that these are crucial factors, and cited locally-relevant angles of each. These are (i) communication: The lack of transparent regulatory guidelines; (ii) resources: The lack of funds; (iii) dispositions: The implementing officials may be unfamiliar with the requirements of specific areas; and (iv) the bureaucracy: In India, health is a state subject, and thus state authorities implement any national health policy, which can lead to complications.

Finally, India can take inspiration from the highly successful French model of a multidisciplinary approach, combined with high standards of safety and quality, tailored for the orphan industry [58]. Along with an OD policy, the French government also created the Fondation Maladies Rares (Rare Disease Foundation) and centers of excellence for RD research such as the Agence Nationale de la Recherche and the Hospitalier de Recherche Clinique.

In summary, we have mapped in detail, the work, goals, challenges, achievements, and recommendations to the government of most of the OMPOs in India. This will help the government to identify areas that need further work, and mechanisms to facilitate the OMPOs’ work in general. Future work could detail the precise supportive measures that the government might take, especially keeping in mind what other countries have done.

Separately, we note that this study has several limitations.

- Sampling: We did not aim to include every OMPO in India in our study. Instead, we aimed for a comprehensive set, which had diverse products or services. Our cohort has excluded a few OMPOs with work that overlaps that of the included OMPOs.
- Unverifiable claims: We had no means to verify the interviewees’ claims.
- The age and gender of each respondent was not taken into account.
- The study was performed at a time when the Indian RD landscape was evolving rapidly, and therefore there may be regulatory or other changes in the near future, which impact the OMPOs.

## Conclusion

Our study aims to draw the attention of policymakers, in particular, to the the work, goals, challenges, achievements, and recommendations of the OMPOs in India. It also emphasizes the urgent need for supportive government policies in this area.

## Supplementary information

**Additional file 1.** Questionnaire used for the interviews.

**Additional file 2.** Details of the interview. The title of each sheet is listed below. Each sheet may contain up to 12 columns, capturing all the questions from the relevant section of the questionnaire. (i) Details of each OMPO, (ii) products or service details, (iii) products or services available to patients, (iv) products in development, (v) production process details, (vi) collaborations, (vii) technical know-how, (viii) government support, (ix) IP issues, (x) challenges.

## Data Availability

The datasets generated and/or analyzed during the study are not available publicly. This was done to keep the interview transcripts confidential. The interview schedule that was used for the interviews is enclosed as Additional file 1. The findings of the interviews have been captured in Additional file 2.

## Abbreviations

AI: Artificial intelligence
APL: Aten Porus Lifesciences
CAGR: Compound annual growth rate
CDD: Center for Drug Discovery
CSIR: Council of Scientific and Industrial Research
DART: Dystrophy Annihilation Research Trust
DCGI: Drug Controller General of India
EMA: European Medicines Agency
FCRA: Foreign Contribution Regulation Act
FSSAI: Food Safety & Standards Authority of India
GLS: Genique Lifesciences
GoI: Government of India
GRAS: Generally recognized as safe
GROW: Genes Repair and Regeneration at Ophthalmic Workstation
GST: Goods and service tax
GUaRDIAN: Genomics for Understanding Rare Diseases - India Alliance Network
IB: Indus Biotech
IEM: Inborn Errors of Metabolism
IoT: Internet of things
NDCTR: New Drugs and Clinical Trials Rules
NIPT: Non-invasive prenatal testing
NN: Narayana Nethralaya
NPD: Niemann-Pick disease
NPTRD: The National Policy for Treatment of Rare Diseases
ODA: The Orphan Drug Act
OMP: Orphan medicinal product
OMPO: Orphan medicinal product organization
POC: Proof of concept
R&D: Research and development
RDs: Rare diseases
RLS: Redcliffe Life Sciences
RP: Retinitis pigmentosa
SMOI: Specialized Mobility Operations and Innovations
TBXT: TerraBlue XT
US: The United States of America
USFDA: The US Food and Drug Administration
WWGM: World Without GNE Myopathy
