# Supplementary material for: The work, goals, challenges, achievements, and recommendations of orphan medicinal product organizations in India: an interview-based study
Source: Orphanet J Rare Dis. 2019 Nov 4;14:241. doi: 10.1186/s13023-019-1224-0 (PMC6829914; doi:10.1186/s13023-019-1224-0)
Supplement: Supplementary file 1 — Additional file 1. Questionnaire used for the interviews. [file 13023_2019_1224_MOESM1_ESM.docx]

**Annexure I.**

Interviewee:

Organization:

Interviewer : Mohua Chakraborty Choudhury

Date :

Time:

Mode:

**Questionnaire for Indian companies involved in rare disease diagnosis, treatment and management in India**

1. **Organization details:**
2. Organization name:
3. Year of starting:
4. Year of starting work specific to a rare disease :
5. Founding members:
6. Name of interviewee:
7. Position/role of the interviewee:
8. Vision, mission and goals of your organization/of rare disease specific activity:
9. Strategy/objectives adopted by the organization:
10. Motive for initiating the work specific to rare disease patients:
11. Initial funding provided by (could be multiple sources):
12. Is there any registry for your rare disease in India, and is your company contributing any data to this registry, or any other database?
13. **Product/service details:**
14. Overall products/services:
15. Products/services that cater specifically to rare disease patients:
16. What is the general strategy for product development in your company? Are you bringing out first-in-class molecules, or follow-ons or generics? Or a combination?
17. Is there any similarity in the drug pathway or action mechanism of your existing products?
18. Did you assess/quantify the demand for the type of service/product that you offer/plan to offer, before planning to do so?
19. If so, how did you do this (through field surveys, for instance)? If so, over what geographies or populations?
20. Has the company pivoted in its product line? Has it changed its strategy.
21. Has your product/service reached the market, or is still in development?
    1. **Products that cater to rare disease patients that have reached the market:**
22. Products/services that cater to rare disease patients:
23. The year of product concept, first patent filed and first sales
24. How long did it take from conception of idea to bring it out to the market. If there was any delay what caused it?
25. How did you define meaningful product specifications?
26. Basic technological approach:
27. What are the competing product/services available in the market? Which are Indian, and which are foreign?
28. Compared to what is in the market, is your product/service better/different in terms of meeting patient needs?
29. Is your product/service more affordable than the available alternatives (Indian/foreign)?
30. If so, comment on the strategy that you adopted to bring a lower priced product to the market (possible strategies: local sourcing, bulk sourcing, innovative design, lower cost of manufacturing, reducing profit margin, anything else).
31. Which regulatory approvals have you received (Indian/US/Europe? DCGI/FDA/CE Mark)?
32. What was the official cost for each approval? What were all the other costs associated with each filing?
33. Is there any system of getting market feedback about the product/service and making improvements when warranted?
34. How do you assure your consumer about the reliability of your products?
35. What kind of challenges did you face in penetrating the market?
36. Have you started earning revenue in India from the rare disease market?
37. Have you targeted any market outside India? If so, which ones?
38. Which markets are you getting maximum traction in?
    1. **Products/services in development that cater to rare disease patients:**
39. How many product development projects have you run/are running? Presumably they are all in India?
40. How did you construct meaningful product specifications? When did you start product/service platform development?
41. Where are you in the process of product/service platform development?
42. Who has funded it?
43. When are you likely to be able to offer it to the patient?
44. What would be the likely price point?
45. How does this compare to what is available in India/abroad and if abroad, then which countries are we referring to?
46. If priced lower, what factors make it lower? (for instance: local sourcing, bulk sourcing, innovative design, lower cost of manufacturing, reducing profit margin, anything else)
47. Which regulatory approvals are you aiming at (Indian/US/Europe? DCGI/FDA/CE Mark)?
48. For each one that you are aiming for, (a) what are the filing costs? (b) What do you think would be all the other costs associated with each filing?
49. Up to which stage do you want to take your products? Out-license it after preclinical/phase 1/phase 2, or take to the market?
50. What are the biggest challenges for your products-in-development?
51. For your locally developed products, do you plan to take them to other markets? If so, some examples of markets which you might target?
52. **Production process details:**

Basic Technologies used:

1. Raw material and consumables:
   - 1. What fraction is imported (roughly)?
     2. Do the imported components form a major or a minor part of the process?
     3. Roughly what fraction of the cost of production do they comprise?
     4. Do they come under capital expenditure or recurrent expenditure?
2. For the fraction that is imported, is an alternate available domestically, and if so is it of adequate quality? Substituting it with an equivalent domestic product could reduce the final cost by roughly how much? If it is not produced locally, what factors do you think would make it so?
3. Instrumentation:
   - 1. Roughly what has been your capex on lab set up/instrumentation?
     2. Did you have to import any instrumentation or other components of your capex?
4. Are there important components used in your production process, which if produced locally, could have served other industries in India/abroad?
5. What are some of the biggest challenges you faced in establishing your production process?

1. **Collaborations:**
2. Did you need local subject matter experts in the area of rare diseases that are your focus? If so, did you face a problem in finding such experts?
3. Do you have any local collaborations? What is the nature of such collaborations? Would you be willing to name the collaborating organization(s)?
4. Did you enter into any clinical development program/ clinical trials? What are the challenges and issues you faced?
5. Do you have a clinical partner? Like hospital, diagnostic center. Would you be willing to name the collaborating organization(s)?
6. Do you have any international partnerships? If so,
   - 1. How were they initiated?
     2. What is the nature of the collaboration in each case?
     3. In case R&D is being done collaboratively, for instance, what is the source of funding?

iv. Would you be willing to name the collaborating organization(s)?

1. What challenges did you face in establishing a productive international collaboration?
2. **Technical know-how:**
3. How was your initial technical know-how developed? Was it developed locally, or developed through an international collaboration?
4. Was it on your own, or through informal advice, or a consultancy, or is there a license or some other arrangement? Were there any financials involved?
5. What percentage of the initial expenditure was spent on technical know-how?
6. Has your technical strategy changed over the years? If so, in what way?
7. What were the challenges that you faced in acquiring your technical know-how?
8. **Government support:**
9. Any incentives available from Government - monetary or otherwise?
10. Tax benefits? If so, for what?
11. Funding? If so, for what?
12. Distribution channel? If so, for what?
13. Challenges in terms of government support and policy:
14. What are the kinds of changes you would like to see in government policy that will help your business/work in particular?
15. What are the kinds of changes you would like to see in government policy that will help your patients in other ways?
16. Could you give some examples of support that you see that rare disease organizations (for-profit or not-for-profit) get from other governments that you do not get in India?
17. **Intellectual property issues :**
18. Have you protected your product with patents, or any other IP?
19. If so, Indian patents/US/PCT route...?
20. Have you in-licensed/out-licensed your IP?
21. If sourced from within India, from which type of organization? Would you be willing to name the organization?
22. In-licensed IP: Have you sourced IP from abroad, and if so, could you talk about it the terms of the license, such as geographic area or subject area...? Exclusive/non-exclusive?
23. Any challenges related to acquiring, protecting or defending your IP?
24. Have you encountered IP theft in India, or are aware of cases in India of such theft? If the latter, would you be willing to give an example?
25. **Challenges:**
26. Could you discuss some of the biggest challenges you have faced in building your organization, especially from the point of view of the rare disease work?
27. Did you face a challenge in market accessibility due to lack of prevalence data or any other reason? If so, what were those reasons?
